# Radiographic Evolution of Contralateral Asymptomatic Incomplete Atypical Femoral Fractures in Autoimmune Disease Patients

**DOI:** 10.3390/diagnostics16020350

**Published:** 2026-01-21

**Authors:** Tomofumi Nishino, Kojiro Hyodo, Yukei Matsumoto, Yohei Yanagisawa, Koshiro Shimasaki, Ryunosuke Watanabe, Tomohiro Yoshizawa, Hajime Mishima

**Affiliations:** Department of Orthopaedic Surgery, Institute of Medicine, University of Tsukuba, 1-1-1 Tennodai, Tsukuba 305-8575, Ibaraki, Japan; pjxgr965@tsukuba-seikei.jp (K.H.); yukeimatsumoto@tsukuba-seikei.jp (Y.M.); yanagisawa@tsukuba-seikei.jp (Y.Y.); koshiro19881020@tsukuba-seikei.jp (K.S.); ryuwatanabe@tsukuba-seikei.jp (R.W.); tyoshizawa@tsukuba-seikei.jp (T.Y.); hmishima@tsukuba-seikei.jp (H.M.)

**Keywords:** atypical femoral fracture (AFF), bisphosphonate (BP), autoimmune disease, glucocorticoid (GC)

## Abstract

**Background/Objectives:** Atypical femoral fracture (AFF) represents a diagnostic and therapeutic challenge, particularly in autoimmune disease patients receiving long-term bisphosphonate (BP) and glucocorticoid (GC) therapy. Although bilateral AFF is common, the radiographic evolution of asymptomatic incomplete lesions identified at the time of a complete fracture remains insufficiently defined. This study aimed to characterize the natural history and imaging biomarkers associated with progression in this biologically homogeneous high-risk population. **Methods:** Ten female autoimmune disease patients with complete AFF and asymptomatic incomplete contralateral lesions were retrospectively evaluated over a mean 59 months. Serial radiographs were assessed for cortical beaking, periosteal flaring, and transverse radiolucent lines. All patients discontinued BP therapy postoperatively; teriparatide was administered when tolerated. **Results:** Six lesions regressed, three remained stable, and one progressed—this progressing case being the only limb with a transverse radiolucent line at baseline. No patient developed symptoms or sustained a complete fracture on the contralateral side. Radiographic remodeling occurred independently of symptoms. BP discontinuation and, when tolerated, teriparatide appeared to contribute to lesion stabilization, although statistical significance was not achieved. **Conclusions:** In autoimmune patients with severe long-term BP and GC exposure, most asymptomatic incomplete AFF identified at the time of contralateral complete fracture remains stable or improves under conservative management. A transverse radiolucent line is the most decisive imaging biomarker predictive of progression and warrants intensified surveillance or consideration of prophylactic fixation. Larger cohorts are needed to refine risk stratification algorithms and optimize diagnostic and management strategies.

## 1. Introduction

Atypical femoral fractures (AFF) are an uncommon yet clinically significant complication associated with long-term antiresorptive therapy. Unlike typical osteoporotic fractures, AFFs are characterized by a high risk of delayed union, nonunion, or pseudarthrosis, particularly when treated after progression to a complete fracture. The American Society for Bone and Mineral Research (ASBMR) Task Force has issued two influential reports recommending proactive management of incomplete AFF, including prophylactic fixation, to prevent conversion to complete fractures and the substantial morbidity associated with them [1,2]. These recommendations underscore the importance of early and accurate radiographic diagnosis.

Although the overall incidence of AFF is low, bilateral involvement is relatively common. Consequently, evaluation of the contralateral femur at the time of a complete fracture provides a valuable diagnostic opportunity. While routine imaging of all patients receiving long-term bisphosphonates (BP) is impractical, presentation with a complete AFF enables efficient screening for asymptomatic incomplete lesions that might otherwise remain undetected until progression occurs.

At our institution, most AFF cases occur in patients with autoimmune diseases who have undergone prolonged treatment with both glucocorticoids (GC) and BP [3]. This represents a distinct and biologically homogenous cohort characterized by severely suppressed bone turnover and a potentially different pattern of AFF development and progression compared with typical osteoporotic populations. Understanding the behavior of incomplete lesions in such high-risk individuals is essential, as long-term GC and BP exposure may synergistically impair cortical bone quality and promote microdamage accumulation.

Despite the clinical importance of early detection, several knowledge gaps remain. First, the natural history of asymptomatic incomplete AFF in autoimmune patients is not well defined, particularly when lesions are identified at the time of a complete contralateral fracture. Second, radiographic markers of progression—such as cortical beaking, periosteal flaring, and transverse radiolucent lines—have not been comprehensively characterized in populations exposed to both GC and BP. Third, the effects of altering bone metabolism, including BP discontinuation and teriparatide administration, on lesion evolution remain unclear in this group. Finally, current ASBMR-based recommendations do not clarify whether all asymptomatic incomplete lesions warrant prophylactic fixation or whether some may remain stable under conservative observation. These uncertainties highlight the need for data describing radiographic behavior in a clearly defined high-risk population.

The aim of this study was therefore to evaluate the long-term radiographic progression of asymptomatic incomplete AFF detected in the contralateral femur at the time of complete fracture in autoimmune disease patients receiving prolonged BP and GC therapy. Given that prior studies and consensus reports have highlighted substantial heterogeneity in AFF populations with respect to underlying disease, pharmacologic exposure, and biomechanical factors, interpretation of radiographic progression remains challenging. By standardizing underlying disease characteristics and long-term exposure to bisphosphonates and glucocorticoids, the present study intentionally reduces biological heterogeneity and enables a more focused analysis of lesion behavior.

## 2. Materials and Methods

### 2.1. Study Population and Case Selection

Between 2009 and 2023, all consecutive patients who underwent surgical treatment for complete AFF at our institution were retrospectively identified from institutional surgical records. All fractures met the diagnostic criteria established by the Second ASBMR Task Force Report [1]. According to this definition, AFF involves a transverse fracture of the femoral diaphysis extending from just below the lesser trochanter to just above the supracondylar flare, requiring at least four of the five major radiographic features: minimal trauma, a transverse lateral cortical fracture line, non- or minimally comminuted morphology, and localized periosteal or endosteal thickening. Minor features—including generalized cortical thickening, prodromal thigh pain, or delayed healing—are supportive indicators. These criteria were strictly applied to ensure diagnostic consistency.

Of these 22 patients, 19 had autoimmune diseases and had been treated with long-term GC and BP. This subgroup was selected intentionally to minimize biological heterogeneity and enhance internal validity, as more than 90% of AFF cases in our previous cohort shared the same pathological background [3]. No cases meeting the ASBMR diagnostic criteria were intentionally excluded at the initial screening stage. Subsequent exclusions were applied systematically according to predefined criteria, including bilateral simultaneous fractures, inability to evaluate the contralateral femur due to implanted hardware, or absence of radiographic evidence of incomplete lesions. After exclusion of one case with bilateral simultaneous fractures and three cases in which the contralateral femur could not be evaluated due to implanted hardware, 15 patients remained eligible for contralateral assessment. Additional exclusions included one patient with symptomatic incomplete AFF requiring prophylactic fixation and four patients lacking radiographic evidence of incomplete lesions. Ultimately, 10 patients with asymptomatic incomplete AFF on the contralateral femur were included in the final analysis (Table 1).

All participants were female (mean age 61 years, range 49–72) with a mean BMI of 24.8 kg/m^2^ (20.4–29.4). The mean follow-up duration was 59 months (18–112). Underlying autoimmune conditions included rheumatoid arthritis (*n* = 5), interstitial pneumonia (*n* = 2), systemic lupus erythematosus (*n* = 2), overlapping myasthenia gravis (*n* = 1), dermatomyositis (*n* = 1), adult Still’s disease (*n* = 1), and IgG4-related disease (*n* = 1). Mean durations of GC and BP therapy were 16 years (9–28) and 8 years (4–20), respectively. Alendronate was the most commonly used BP; eight patients received it at some point, and three patients switched BPs during treatment. Lesion laterality was evenly distributed across right and left femurs.

Incomplete lesions were located in the subtrochanteric region in eight limbs and the diaphyseal region in two limbs, and all mirroring the fracture sites on the operative side—an observation consistent with prior reports of bilateral symmetry in AFF. None of the patients demonstrated lateral femoral bowing as a potential biomechanical contributor [4]. Radiographic characteristics at baseline included cortical beaking (*n* = 6), periosteal flaring (*n* = 4), and a transverse radiolucent line (*n* = 1). Both flaring and a radiolucent line were present in the latter case (case #2), representing a high-risk morphology.

### 2.2. Treatment Protocol and Radiographic Evaluation

Following surgical management of the complete AFF, BP therapy was discontinued in all cases. Teriparatide was initiated beginning with the fourth case and continued unless adverse effects occurred. All patients underwent standardized radiographic follow-up using anteroposterior and lateral femoral views at predetermined intervals. Radiographs obtained at injury and at the final follow-up were compared to evaluate. While the ASBMR major criteria were applied for diagnostic inclusion, serial radiographic evaluation in this study focused on a predefined subset of morphological features relevant to the progression of incomplete lesions. These features were selected a priori based on their reported association with lesion instability and included cortical beaking, periosteal flaring, and transverse radiolucent fracture lines:

- Widening or reduction of cortical beaking;
- Changes in flaring morphology;
- Appearance or progression of radiolucent fracture lines;
- Development of new incomplete fracture features.

These radiographic outcomes were operationalized a priori to improve reproducibility and reduce evaluator subjectivity—an approach encouraged by diagnostic research methodology.

Lesion evolution (progression, stability, or regression) was independently assessed by three treating orthopedic surgeons (TN, YM, YY). Discrepancies were resolved by consensus, enhancing interobserver reliability, a key point for methodological rigor in imaging-based studies. The purpose of independent assessment was to minimize individual interpretative bias prior to establishing a final consensus-based classification, which served as the reference standard for analysis. Given the small sample size and the categorical nature of lesion evolution, formal inter-rater agreement statistics such as kappa coefficients were not calculated, as such measures would be statistically unstable and potentially misleading. Notably, no major discrepancies occurred that altered the final consensus classification.

Parallel evaluation of the healing course of the complete fracture on the operative side was also performed, including union time and the need for secondary procedures.

### 2.3. Statistical Analysis

Multivariate analysis was performed with lesion extension as the objective variable, and age, BMI, fracture site, comorbidity, GC duration, BP duration, and teriparatide as explanatory variables using JMP 14.3.0 software (SAS Institute, Cary, NC, USA). The significance level, *p*-value, was set at 0.05. Given the limited cohort size, multivariate analysis was performed in an exploratory, hypothesis-generating manner rather than for confirmatory statistical inference. This analysis was intended to assess potential directional associations between lesion behavior and biological or treatment-related variables, and to complement descriptive and longitudinal radiographic evaluation rather than to serve as a standalone determinant of significance.

### 2.4. Ethical Considerations

All patients provided informed consent for data use and publication. This retrospective study was approved by the Institutional Review Board (approval code: H27-041) and conducted in accordance with the Declaration of Helsinki.

## 3. Results

The overall radiographic and clinical outcomes of the cohort are summarized in Table 2. Importantly, throughout the observation period, none of the contralateral incomplete lesions became symptomatic. This finding highlights that radiographic progression of incomplete AFF may occur in the absence of thigh pain or new symptoms, rather than implying that clinical symptoms are irrelevant to surgical decision-making in complete AFF.

**Figure 1:**
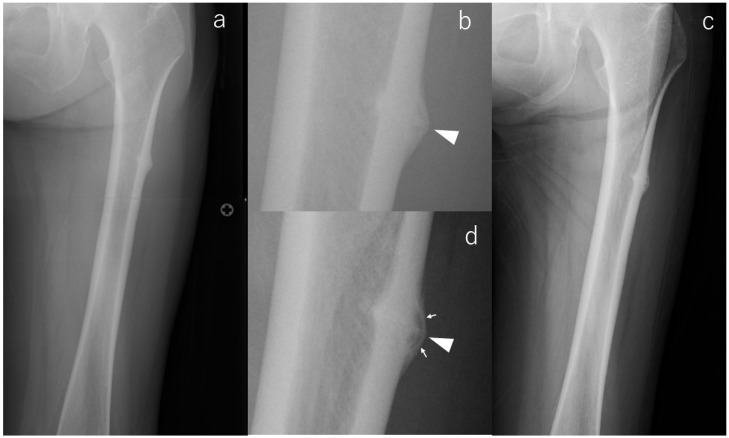
X-rays of case #2 at various time points. (**a**): Radiograph at the time of injury, (**b**): Enlarged image of a; radiolucency running across the cortex (arrowhead), (**c**): Radiograph at the last observation, (**d**): Enlarged image of (**c**); magnified fracture line (arrowhead) and radiolucency in the beak (arrows).

Consistent with the concept that radiographic morphology can progress silently, one limb demonstrated clear enlargement of the incomplete lesion, corresponding to case #2 (Figure 1). At baseline, radiographs of the contralateral femur revealed pronounced subtrochanteric lateral cortical bulging accompanied by beaking and a transverse radiolucent line—findings known to represent a higher-risk diagnostic phenotype. Eighteen months later, follow-up imaging showed a marked increase in beaking size and well-defined internal radiolucency, indicating radiographic progression despite the absence of symptoms. Because the patient died from malignancy 18 months after surgery, extended imaging follow-up was not possible.

**Figure 2:**
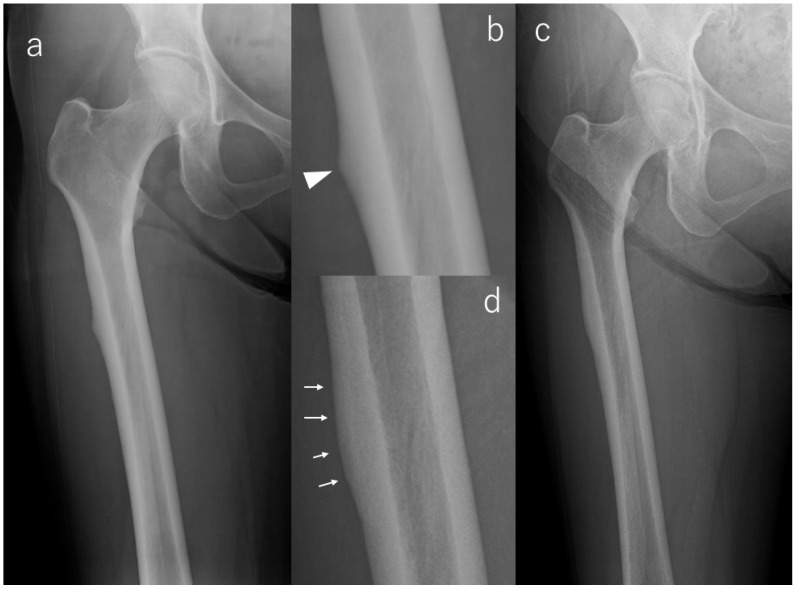
X-rays of Case #3. (**a**): Radiograph at the time of injury, (**b**): Enlarged image of a; bump (arrowhead) in the lateral cortex, (**c**): Radiograph at the last observation, (**d**): Enlarged image of (**c**); flattening of beak tip (arrows).

In contrast, a majority of limbs demonstrated favorable radiographic evolution. Lesion reduction was observed in six limbs, with three showing notable flattening of cortical beaking and three demonstrating decreased periosteal flaring. All of these improvements occurred without symptom onset, emphasizing that radiographic remodeling may proceed independently of clinical manifestations. Case #3 serves as a representative example (Figure 2): initial postoperative imaging demonstrated a lateral subtrochanteric cortical prominence without a fracture line, which progressively flattened over 112 months of surveillance. This improvement was already visible at 36 months and remained stable thereafter, with no radiolucent line ever appearing.

For three additional limbs, lesion morphology remained radiographically stable, reflecting a subgroup in which incomplete AFF may persist without progression when biological and mechanical risk factors remain controlled.

Regarding pharmacologic management, teriparatide was administered in six patients, while four patients did not receive it due to intolerance or treatment timing. Among patients who did not receive teriparatide, one lesion showed radiographic progression and three demonstrated improvement. Given the small sample size and non-randomized treatment allocation, no causal inference regarding the effect of teriparatide on lesion behavior can be drawn from these observations.

Healing of the complete fracture on the operative side was achieved in six limbs, with a mean union time of 9 months (range, 6–16 months). Pseudarthrosis developed in four limbs, necessitating additional surgical intervention in three. These procedures included hemiarthroplasty with a hook plate at 7 months in case #5, plate-assisted nail replacement with bone grafting at 18 months in case #7, and dynamization of the intramedullary nail in case #8 [5] through removal of the distal screw at 31 months. Notably, only case #7 adhered to a postoperative unloading protocol, whereas the remaining patients were allowed full weight bearing, suggesting that mechanical factors alone did not dictate healing outcomes.

When operative-side healing complications were examined in relation to contralateral lesion behavior, no consistent pattern was identified. Among the four patients who developed pseudarthrosis on the operative side, contralateral incomplete lesions demonstrated either radiographic regression or stability, and none progressed to complete fracture. Conversely, the single case of contralateral lesion progression occurred in a patient without confirmed nonunion-related mechanical overload at the time of observation.

Finally, a multivariate analysis evaluating lesion extent as the dependent variable found no significant explanatory factors, likely reflecting the small cohort size and the complex interplay of biological and pharmacological influences in this highly specialized patient population. These findings reflect the limited statistical power of the cohort and support the interpretation that lesion behavior in this study is more appropriately characterized through individual longitudinal imaging patterns rather than through statistical modeling.

## 4. Discussion

Asymptomatic contralateral femoral lesions in patients with AFF are frequently underrecognized until they progress to complete fractures [6]. This underdiagnosis reflects both the subtlety of early radiographic abnormalities and the absence of symptoms in many patients. In complex clinical settings, particularly among individuals with autoimmune diseases undergoing long-term BP and GC therapy, the optimal duration of surveillance and the radiographic markers most predictive of lesion progression remain insufficiently defined.

Probyn et al. demonstrated that among 124 patients with AFF receiving BP therapy, contralateral AFF was detected within 12 months in 62.9% and within 3 years in 88.5% of cases, with bilateral lesions often showing similar anatomic locations and radiographic morphology [7]. These findings highlight the need for systematic contralateral imaging at the time of complete fracture, even in the absence of symptoms.

Lee et al. further advanced this concept by stratifying 53 contralateral limbs into radiographic grades based on early imaging biomarkers. Their Grade 2 group—characterized by beaking, flaring, or a transverse radiolucent line—exhibited a markedly higher rate of progression than Grade 1 (53.6% versus 12%), with a mean time to progression of 25.6 months. Continued BP exposure was a significant accelerator of radiographic worsening [8]. These results underscore the diagnostic value of specific radiographic markers and support the need for at least two years of imaging follow-up even in asymptomatic patients.

Our findings corroborate these observations: the only progressing lesion in our study was also the only case with a transverse radiolucent line at baseline, emphasizing the potential role of radiolucent lines as a high-risk imaging biomarker. Notably, although most incomplete lesions remained stable or regressed following BP discontinuation, the presence of a radiolucent line appears to signify ongoing mechanical and biological vulnerability even after antiresorptive therapy is withdrawn. This radiographic phenotype warrants heightened surveillance and may justify early prophylactic fixation despite the absence of pain.

Conversely, prior literature and clinical guidelines generally recommend prophylactic fixation for incomplete AFFs that are symptomatic or radiographically progressive. Lee et al. reported that nearly half of incomplete AFFs ultimately required surgery, with subtrochanteric location representing a significant risk factor [9]. Although our cohort did not demonstrate clear location-based differences—likely due to small sample size—eight of the ten lesions were subtrochanteric, consistent with previous descriptions of the characteristic distribution of AFF.

The prognostic significance of radiolucent lines has been highlighted by Saleh et al., who found that only 2 of 9 limbs with radiolucent lines healed conservatively, while 7 required surgery within approximately 3 months [9]. In contrast, all limbs without radiolucent lines healed with conservative management. Our finding that the only radiolucent-line lesion was also the only progressing case strongly aligns with their conclusions and reinforces the role of radiolucent lines as a critical imaging biomarker for risk stratification.

Min et al. proposed a scoring system incorporating pain, fracture location, contralateral status, and radiographic findings, recommending prophylactic surgery for scores ≥8 [10]. In our series, the diaphyseal lesions scored 5 and the subtrochanteric lesions scored 6 due to absence of pain and the presence of a complete fracture contralaterally—factors that reduce the overall risk score. These scores correspond to conservative rather than surgical management, which was reflected in our results: one lesion exhibited radiographic enlargement, but none progressed to complete fracture. The presence of a complete fracture on the opposite side may have heightened clinical vigilance and patient adherence, indirectly contributing to the stability observed in most cases.

All patients in this cohort had autoimmune diseases and received prolonged GC and BP therapy, a combination known to suppress bone turnover severely [11]. GC exposure further disrupts bone metabolism by increasing osteoclast activity, suppressing osteoblast differentiation, and accelerating bone cell apoptosis. Sato et al. reported that 8% of autoimmune disease patients receiving GC and BP exhibited cortical beaking [12], consistent with our cohort’s radiographic profile but with less frequent lesion progression. Surgical stabilization on the contralateral side may have contributed to this difference by reducing mechanical stress on the incomplete lesion.

Although teriparatide has been reported to promote fracture healing in atypical femoral fractures [13,14,15], its effect on asymptomatic incomplete lesions remains uncertain. In the present cohort, the small number of patients and the absence of a controlled comparison preclude definitive conclusions regarding its efficacy. Importantly, radiographic stabilization or regression was also observed in patients managed with bisphosphonate withdrawal alone, suggesting that cessation of antiresorptive therapy may represent a key baseline intervention.

These observations suggest that delayed union or pseudarthrosis on the operative side did not systematically translate into increased mechanical or biological stress sufficient to alter the radiographic course of asymptomatic contralateral incomplete lesions in this cohort.

The pathogenesis of AFF is multifactorial, reflecting both biological and biomechanical influences. Given the autoimmune background and uniform exposure to GC and BP in our cohort, biological factors likely dominated lesion evolution. Our findings highlight the importance of imaging biomarkers—particularly beaking, flaring, and radiolucent lines—in understanding lesion behavior and guiding clinical management in such biologically high-risk patients. It should be emphasized that the absence of symptoms in contralateral incomplete lesions does not contradict established indications for surgical treatment of complete AFF, but rather underscores the diagnostic importance of radiographic surveillance in asymptomatic limbs.

This study demonstrates the value of long-term follow-up in clarifying the natural history of asymptomatic incomplete AFF in autoimmune patients. Although teriparatide’s effect remains inconclusive, our findings support its continued investigation. Ultimately, treatment strategies that integrate both biological and biomechanical perspectives will be necessary to improve patient outcomes.

The primary limitation of this study is the small sample size. Accordingly, the absence of statistically significant findings in multivariate analysis should be interpreted in the context of limited statistical power, and the primary value of this study lies in its detailed descriptive and longitudinal assessment of individual lesion trajectories rather than in inferential modeling. Additionally, our institution treats a disproportionately high number of patients with collagen diseases, resulting in a cohort with >90% long-term GC and BP exposure. While this may limit generalizability to broader AFF populations, it provides a unique opportunity to study AFF pathophysiology in a biologically homogenous high-risk group. Larger multicenter cohorts will be essential to validate these findings and refine diagnostic and therapeutic algorithms.

Although the present study shares certain characteristics with a descriptive case series, this design enabled detailed, long-term, lesion-specific radiographic tracking within a biologically homogeneous high-risk population. From a diagnostic imaging perspective, such focused longitudinal assessment provides insights into morphology-driven risk that may be obscured in larger but more heterogeneous cohorts.

## 5. Conclusions

In this biologically homogeneous cohort of autoimmune disease patients with long-term bisphosphonate and glucocorticoid exposure, most asymptomatic incomplete atypical femoral fractures identified at the time of contralateral complete fracture demonstrated radiographic stability or spontaneous improvement following bisphosphonate withdrawal.

A transverse radiolucent line emerged as the most clinically meaningful imaging biomarker predictive of progression and should prompt heightened surveillance or consideration of prophylactic fixation, even in the absence of symptoms. Although the effect of teriparatide could not be clearly established, biological plausibility supports further investigation.

While bisphosphonate withdrawal appears to play a central role in lesion stabilization, the contribution of teriparatide could not be conclusively determined and should be regarded as biologically plausible but unproven in this setting.

Given the small sample size, larger multicenter studies are needed to refine risk stratification frameworks and establish evidence-based diagnostic and management algorithms for incomplete AFF in autoimmune disease patients.

## Figures and Tables

**Table 1:** Patient demographics and status of incomplete fracture.

#	Age (yrs)	Gender	Body Mass Index (kg/m^2)^	Follow-Up Period (mo)	Comorbities	Duration of GC Use (yrs)	Kind of BP	Duration of BP Use (yrs)	Affected Side	Location	Radiographic Findings		
											Beaking	Flaring	Radiolucent Line
1	72	Female	29.4	60	Myasthenia gravis	28	Risedronate	5	Left	Subtrochanter	−	+	−
2	54	Female	24.5	18	Dermatomyositis, Interstitial pneumonia	18	Alendronate	4	Left	Subtrochanter	−	+	+
3	54	Female	25.0	112	Rheumatoid arthritis	9	Alendronate	5.5	Right	Subtrochanter	+	−	−
4	50	Female	20.4	90	Rheumatoid arthritis	20	Alendronate/Minodronate	6.5	Right	Femoral diaphysis	+	−	−
5	79	Female	23.5	75	Adult Still’s disease	11	Etidronate/Alendronate	7	Right	Subtrochanter	−	+	−
6	67	Female	27.1	74	Interstitial pneumonia	9	Alendronate	9	Right	Subtrochanter	−	+	−
7	73	Female	24.4	46	Systemic lupus erythematosus, Rheumatoid arthritis	20	Etidronate/Alendronate	12	Left	Subtrochanter	+	−	−
8	58	Female	29.1	43	Rheumatoid arthritis	19	Alendronate	19.5	Right	Subtrochanter	+	−	−
9	56	Female	24.4	41	IgG4-related disease	10	Alendronate	10	Left	Femoral diaphysis	+	−	−
10	49	Female	20.6	34	Systemic lupus erythematosus, Rheumatoid arthritis	11	Minodronate	5.5	Left	Subtrochanter	+	−	−

#2: death while follow-up. #1&3: change hospital while follow-up.

**Table 2:** Treatment, opposite limb status and results.

#	Location	Completion of Teriparatide Use	Duration of Bone Union/Non-Union in Opposite Side	Lesion Change	Change Details
1	Subtrochanteric	−	6 months	Shrinkage	Flare reduction
2	Subtrochanteric	−	10 months	Enlargement	Fracture line appearance
3	Subtrochanteric	−	16 months	Shrinkage	Beak flattening
4	Femoral diaphysis	+	10 months	Shrinkage	Beak flattening
5	Subtrochanteric	−	Non-union (atrophic) *^1^	Shrinkage	Flare reduction
6	Subtrochanteric	+	6 months	Shrinkage	Flare reduction
7	Subtrochanteric	+	Non-union (atrophic) *^2^	No change	-
8	Subtrochanteric	+	Non-union (atrophic) *^3^	Shrinkage	Beak flattening
9	Femoral diaphysis	+	6 months	No change	-
10	Subtrochanteric	+	Non-union (atrophic)	No change	-

* Additional surgery required. 1: Replacement into prosthesis at 7 months. 2: Nail exchange with plating and bone graft at 18 months. 3: Dynamization at 31 months.

## Data Availability

The data presented in this study are available on request from the corresponding author due to ethical restrictions and the need to protect patient privacy.

## Abbreviations

The following abbreviations are used in this manuscript:

AFF	Atypical femoral fractures
ASBMR	The American Society for Bone and Mineral Research
BP	bisphosphonates
GC	glucocorticoids
